# The peptide PROTAC modality: a novel strategy for targeted protein ubiquitination

**DOI:** 10.7150/thno.46985

**Published:** 2020-08-08

**Authors:** Jinmei Jin, Ye Wu, Jinjiao Chen, Yiwen Shen, Lijun Zhang, Hong Zhang, Lili Chen, Hebao Yuan, Hongzhuan Chen, Weidong Zhang, Xin Luan

**Affiliations:** 1 Institute of Interdisciplinary Integrative Medicine Research, Shuguang Hospital, Shanghai University of Traditional Chinese Medicine, Shanghai 201203, China.; 2 Department of Pharmacology, School of Pharmacy, Fudan University, Shanghai 201203, China.; 3 Department of Pharmaceutical Sciences, College of Pharmacy, University of Michigan, Ann Arbor, MI 48109 US.; 4 Department of Pharmacology and Chemical Biology, Shanghai Universities Collaborative Innovation Center for Translational Medicine, Shanghai Jiao Tong University School of Medicine, Shanghai 200025, China.

**Keywords:** peptide PROTAC, ubiquitination, undruggable proteins, E3 ligase

## Abstract

Despite dramatic advances in drug discovery over the decades, effective therapeutic strategies for cancers treatment are still in urgent demands. PROteolysis TArgeting Chimera (PROTAC), a novel therapeutic modality, has been vigorously promoted in preclinical and clinical applications. Unlike small molecule PROTAC, peptide PROTAC (p-PROTAC) with advantages of high specificity and low toxicity, while avoiding the limitations of shallow binding pockets through large interacting surfaces, provides promising substitutions for E3 ubiquitin ligase complex-mediated ubiquitination of “undruggable proteins”. It is worth noting that successful applications of p-PROTAC still have some obstacles, including low stability and poor membrane permeability. Hence, we highlight that p-PROTAC combined with cell-penetrating peptides, constrained conformation technique, and targeted delivery systems could be the future efforts for potential translational research.

## Introduction

PROteolysis TArgeting Chimera (PROTAC) is an emerging class of therapeutic modality to induce the dynamic degradation of intracellular or nuclear protein of interest (POI), and it plays a significant role in solving drug resistance through the degradation of the entire pathogenic proteins without compensatory increase or mutation 1. For instance, more than 80% of patients with chronic lymphocytic leukemia (CLL) developed C481S mutation after receiving the Bruton's tyrosine kinase (BTK) inhibitor ibrutinib, leading to acquired drug resistance 2. It is gratifying that a series of PROTACs (MT-802, SJF620, and L18I) effectively degrade a variety of clinical BTK mutant proteins and overcome the resistance to ibrutinib induced by BTK mutations 2-4.

The core concept of PROTAC is that this bifunctional molecule binds the POI at one end while binding an E3 ligase at the other end, which forms a ternary complex to hijack the cellular ubiquitin-proteasome system (UPS) for proteasomal degradation of POI 3-5. Compared with small molecule inhibitors, PROTAC modalities usually exhibit improved therapeutic effects due to their enhanced regulation of related fundamental signaling pathway and minimized drug resistance 6. As a vital direction for novel drug discovery, the therapeutic strategies of PROTAC have been successfully applied to conditionally degrade plenty of POIs *in vitro* and *in vivo*, such as estrogen receptor (ER) 7, androgen receptor (AR) 8, bromodomain-containing protein 4 (BRD4) 9-11, anaplastic lymphoma kinase (ALK) 12, 13, Focal adhesion kinase (FAK) 14, cyclin dependent kinase 9 (CDK9) 15, BCR-ABL1 16, 17, and cycle-related and expression-elevated protein in tumor (CREPT) 18. The oral PROTACs ARV-110 for AR (ClinicalTrials.gov Identifier: NCT03888612) and ARV-471 for ER (ClinicalTrials.gov Identifier: NCT04072952) exhibited excellent therapeutic efficacies in preclinical studies, and are currently undergoing Phase I clinical trials for evaluation of their safety and tolerability. The preliminary clinical data of ARV-110 exhibited good safety and efficacy in patients with metastatic Castration-resistant Prostate Cancer 19.

However, most of the current PROTACs are using small molecules as targeting warheads, which heavily rely on the binding pockets of POI 20. With the rapid development of structural biology, it is coming along more convenient to obtain the peptides with high affinity to POI epitopes 21, 22. Therefore, designing PROTAC based on the specific peptides (p-PROTAC) is an emerging approach to realize the specific and effective degradation of POI, and extend the scope in regards to “undruggable” proteins targeting, while avoiding the restriction of shallow binding pockets through large interacting surfaces between POI and peptides 4, 23. Compared with small molecule PROTACs, p-PROTACs have exhibited several unique advantages (**Table 1**). The previously reported peptide targeting warheads of p-PROTACs and E3 ubiquitin ligase-recruiting ligands so far were endogenous peptides with high safety and affinity. Those endogenous ligands have been regarded as ideal choices for drug development compared with other modalities (small molecules and antibodies) 24. However, it is worth noting that most of the subsequent researches chose small molecule ligands due to the poor permeability and low stability of conventional peptides. Hence, the multifaceted understanding of p-PROTAC should be highly valued which can further promote its potential development.

In this review, we summarized the proven approaches for design, synthesis, and application of p-PROTAC, while highlighting their unique characteristics and advantages. Regarding the current bottlenecks for clinical translation of p-PROTAC, we also especially focus on the potential efforts in establishing p-PROTAC platforms on the grounds of interdisciplinary technologies.

## Design and synthesis of p-PROTAC

**Figure 1:**
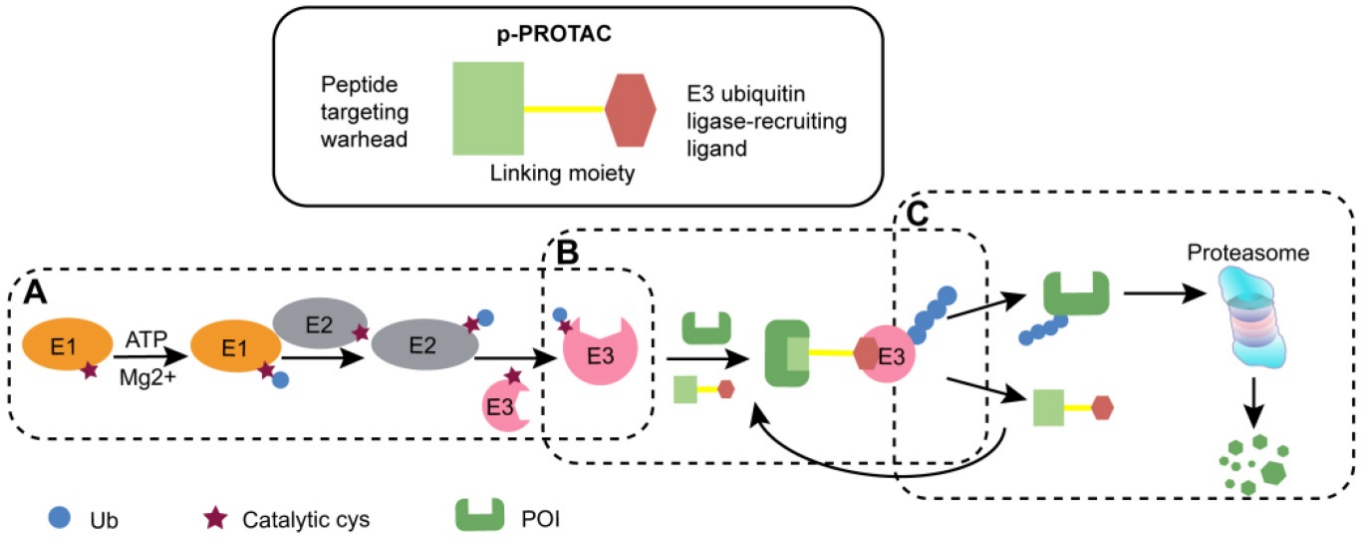
** The schematic diagram of p-PROTAC. (A)** With ATP, the enzyme E1 adheres to the Cys residue of the ubiquitin molecule. Then E1 transfers the activated ubiquitin molecule to the E3 ubiquitin ligase via the ubiquitin-binding enzyme E2. **(B)** Bifunctional p-PROTAC molecule binds to the POI with one end while the other end binding to an E3 ligase to form ternary complex. **(C)** Different types of E3 enzymes jointly recognize POI with polyubiquitinated modification to mediate degradation through ubiquitin proteasome, and then freed p-PROTAC can be recycled.

PROTAC is generally regarded as a bifunctional molecule that acts as a bridge between the POI and E3 ligase to induce the subsequent degradation of POI. p-PROTAC consists of following components, a peptide-based targeting warhead, a chemical linker, and a recruitment ligand for E3 ubiquitin ligase (**Figure 1**) 25-27. In this part, we mainly summarized the design principles for the three components, the strategy to synthesize p-PROTAC, optimization of subsequent peptides, as well as providing theoretical basis for the subsequent use of p-PROTAC.

### Design of p-PROTAC

#### Design of peptide targeting warhead

**Figure 2:**
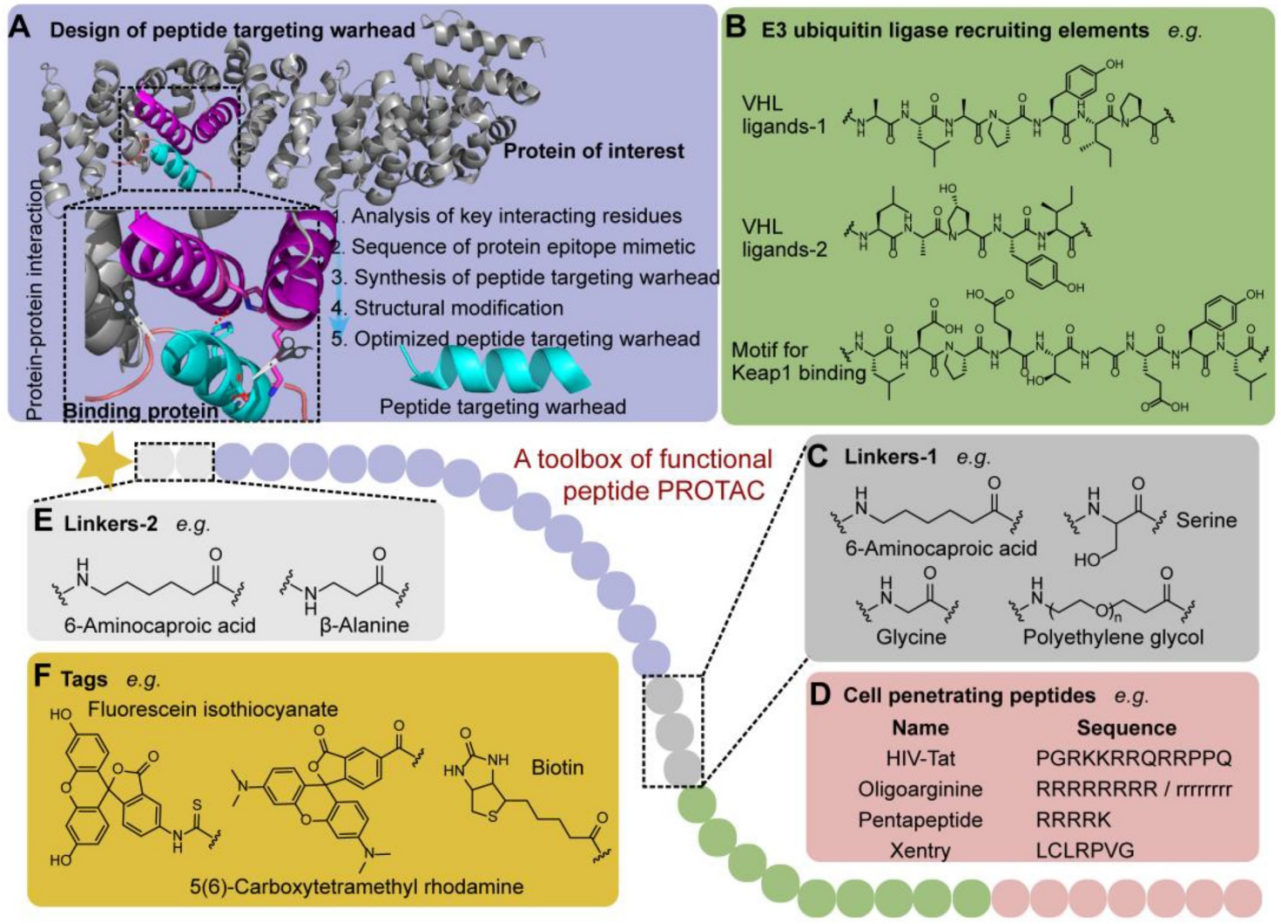
** A toolbox of functional peptide PROTACs. (A)** The design of peptide targeting warhead using chemical epitope targeting strategy (PDB code: 1QZ7). **(B)** The commonly used E3 ligase ligands. **(C)** Linkers-1 connecting POI and E3 ligase ligand. **(D)** Cell penetrating peptides used to increase membrane permeability. **(E)** Linkers-2 connecting POI and tag.** (F)** Functional tags used in p-PROTAC.

The appropriate choice of peptide targeting warheads has appreciable impacts on the binding affinity and spatial orientation of POI and E3 ligase, which affects the ubiquitination efficiency 28. The obtainment of the targeting peptide is not a tedious trial-and-error process like small molecules which extremely relies on the database and virtual screening. Generally, based on the crystal structure of endogenous complex of POI and binding protein which reveals their key protein-protein interaction (PPI) motif and residues (**Figure 2**), the peptide targeting warheads, binding to the POI selectively, can be designed to disrupt the PPI. Then, the obtained sequence of protein epitope mimetic can be used as the leading candidate to synthesize targeting warheads 29. To maximize the efficacy of protein epitope mimetics, the point mutation on the non-critical interacting residues can be further used to optimize peptides targeting warhead with high affinity for a specific POI 29.

For example, using chemical epitope targeting strategy, Leduc et al. 29 proposed the peptide sequences that mimic binding helix of the coactivator domain with the ERα could function as ER antagonists. To maintain α-helical structure and specificity of protein epitope mimetic in short peptide, they reserved consensus pentapeptide motif, and developed a series of helix-stabilized cyclic peptides as selective inhibitors to bind ER tightly and selectively by regulating ER-coactivator interactions. Inspired by these peptidomimetic estrogen receptor modulators (PERMs), Li et al. 30 further used the N-terminal aspartic acid cross-linking strategy (terminal aspartic acid, TD) to design more stabilized peptide modulators (TD-PERMs) with good cell permeability. Then, they 31 conjugated TD-PERMs with the recruiting peptide of the Von Hippel-Lindau (VHL) E3 ligase to form a p-PROTAC molecule with enhanced biological activity compared to TD-PERMs.

In addition, the phage display and yeast display techniques can be used to develop targeting peptides with high affinity to POI 32, 33. These techniques provide alternative tools to obtain peptide targeting warheads with highest frequency clone after several rounds of biopanning. More importantly, these techniques are independent of protein crystal complexes, and D-configuration peptide targeting warheads can be obtained via mirror-image phage display which can avoid easy degradation of L-peptides by proteolytic enzymes *in vivo* 34. We expect that the peptide phage display techniques could be the future trends to design targeting peptides for p-PROTAC.

#### Exploitation of E3 ubiquitin ligase-recruiting ligand

Since ubiquitination tags guide the degradation caused by proteasomes which are hijacked by p-PROTAC to promote the degradation of POIs 35, 36, it is very important to rationally choose and design the recruiting moiety. There are many E3 ubiquitin ligases encoded in our bodies with specific degron recognition motifs 37, which provide huge theoretical possibility for PROTAC drug development. However, only less than 1% of E3 ligases, including Von Hippel-Lindau (VHL), Cereblon (CRBN), IAPs, Keap1, RNF4, RNF114, and MDM2, can be hijacked by PROTAC *in vivo* 38-40. Heretofore, most of reported PROTACs have chosen VHL or CRBN as E3 ligase due to the existence of their specific and high affinity ligands 11, 41-43.

In the first reported PROTAC strategy, the researchers found that a phosphopeptide (DRHDSGLDSM) within IκBα, regulating the recruitment of Skp1-Cullin-F boxβ-TRCP (SCFβ-TRCP) in E3 ubiquitin ligase complexes, can be successfully used to induce the ubiquitination dependent degradation of POI 25. In tumor sites, hypoxia-inducible factor-1α (HIF-1α) can be degraded through the VHL-mediated ubiquitination 44. Therefore, the minimum recognition sequence (ALAPYIP) of HIF-1α has been chosen as an E3 ubiquitin ligase recruiting ligand to induce the degradation of HIF-1α *in vivo* 45, 46. Afterwards, a series of peptides have been further optimized for VHL recruitment, including HIF-1α octapeptide and other five amino acids (LAP(OH)YI) 47. So far, the E3 ubiquitin ligase recruiting ligands for VHL have also been widely applied to design p-PROTAC 18, 31, 33, 48, 49.

Owing to the convenient and easy synthesis, a series of small molecules with the E3 ubiquitin ligase-recruiting function could also be coupled with the targeting warheads to form the p-PROTAC. For example, three amine drugs (thalidomide, lenalidomide, and pomalidomide) have been identified as CRBN ligands 50.

#### Choice of linking moiety

Linking moiety has significant influences on the stability of the ternary complex and subsequent function 39, 51. To maintain the delicate balance between the affinity and the spatial effect, the linking moiety should have an appropriate stereochemical structure and provide a suitable solvent-exposed position to connect the peptide targeting warhead with the E3 ubiquitin ligase-recruiting ligand 52. So far, amino acids are the commonly used linking moieties in p-PROTAC, including aminohexanoic acid (Ahx), glycine, and serine 45, 53. Polyethylene glycol (PEG) is another linking moiety regularly used to improve the hydrophilicity of p-PROTAC. For instance, VHL-recruiting PROTAC targeting ER showed optimal efficiency with 16 atoms chain length between E3 recognition moiety and warhead 54. Interestingly, this PROTAC exhibited improved efficiency and affinity to ER due to the change of linker and connection mode in a separate study 55. Similarly, a series of VHL-recruiting PROTACs targeting FAK exhibited different POI degradation potential due to the different length and constitution of linking moieties 14.

### Synthesis strategy

The solid-phase synthesis strategies with Fmoc-protecting group have been widely used in p-PROTAC synthesis. During the process, the side chains of regular amino acids are protected by an unstable anti-acid protective group, while the α-amino group is protected by an unstable anti-base Fmoc-protective group 56. The selection of resin is determined by the functional groups of C-terminal: the 2-cl-trt or Wang resin are suitable for the retain of C-terminus, while the Rink Amide-AM resin is suitable for the amination of C-terminus 57. The final p-PROTAC products can be separated from the resin and further purified. Compared with the classic organic synthesis and segments linking of molecule, the amino acid condensation is more concise. In addition to the previously mentioned solid-phase synthesis method, the package of those three parts can also be performed with liquid-phase synthesis 25.

As the targeting warhead, peptides possess greater potential in structural modification compared with small molecules 31. For example, the length, type, and the sequence of amino acids can be changed for specific secondary structures and physicochemical properties 58, and the progress of solid-phase synthesis technique further promotes automatic synthesis of peptides which accelerate additional layer of convenience for potential targeting peptide library and p-PROTAC fabrication.

In addition, the fluorescent tags (FITC and rhodamine) can be utilized to track the cell uptake process and fluorescence localization of p-PROTAC 56. The fusion affinity tags (biotin, etc.) can help evaluate the targeting behavior of p-PROTAC by immunoprecipitation or Pull-down assays 54, 57, 59. Non-radionuclide labeling, phosphopeptide synthesis, and other modification methods are also applied in peptide modification 49. However, we must pay special attention to the modification position so that the binding affinity of p-PROTAC to POI and E3 ligase, as well as the stability of the ternary complex should not be affected.

## Characterization of biophysical properties

According to the construction features of p-PROTAC ternary complexes, the following biophysical properties mainly include the determination of conformation, binding affinity with POI, cell membrane permeability, and the characterization of therapeutic effects. More importantly, the ubiquitin-proteasome-dependent degradation process of POI should to be verified carefully. Herein, we present all the techniques used to evaluate these characteristics of p-PROTAC.

### Conformation

In the p-PROTACs, peptides with an α-helical structure and rich positive charge possess better cell membrane permeability 60, 61. The peptides containing an α-helix exhibit the typical absorption peaks at 208 nm and 222 nm in circular dichroism spectrum. At given concentration of the peptides, the α-helix degree of the peptides can be evaluated through the absorption signal strength of the circular dichroism spectrum at 222 nm, which could be utilized to compare the cell membrane permeability and stability of different p-PROTACs. In a recent study, a facile N-terminal aspartic acid cross-linking strategy (TD strategy) was invented to construct p-PROTAC with α-helix 31. Using circular dichroism spectroscopy, they successfully evaluated the helicity of these peptides and proved that the obtained p-PROTAC with α-helix from TD strategy exhibited better cell penetration than linear ones 31.

### Target binding affinity

Fluorescence polarization (FP), isothermal titration calorimetry (ITC), surface plasmon resonance (SPR), microscale thermophoresis (MST), and co-immunoprecipitation (Co-IP) techniques have been widely used to determine the affinity of polypeptides with POI. Even better, the FP, ITC, SPR, and MST can provide the binding constant (K_d_) between them. The characteristics of those binding affinity analysis methods have been listed in **Table 2.**

In the FP assays, the small molecules can be used as fluorophores to detect the formation of complexes from the increased fluorescence polarization for assessing the strength of PPI 62-64. For p-PROTAC strategy, FP is often used to detect the affinity between a polypeptide fragment and POI 31, 48. Previous research found that FP assay could be used to detect the affinity of the fluorescein isothiocyanate (FITC)-labeled peptides with the ERα ligand binding domain, providing varying affinity parameters between different PERM and ERα 31.

ITC is a well-established thermodynamic dependent technique that determines a series of thermodynamic parameters for bimolecular interactions, including binding constants, molar binding enthalpy (Δ*H*°), the binding reaction entropy (Δ*S*°), and the like 65. When constructing p-PROTAC's degradation ability on intracellular Tau, the results of ITC experiments exhibited that peptide 1 retained its binding affinity to Keap1 and Tau, with K_d_ values of 22.8 nM and 763 nM, respectively 26. However, the ITC method also has some obstacles for biological samples evaluation, such as low sensitivity and relatively large number of samples required for sufficient thermal signal.

SPR biosensor technology is widely used because of its rapid selection of fragments with binding affinity. It enables researchers to quantify the binding properties of a lead compound to its target based on affinity, specificity, and association/dissociation rate 66. Due to the directly measured interaction between peptides and POI, the kinetic process of binding and the kinetic data of the interaction can be easily obtained. It should be noted that the modes of surface-immobilized binding partners may affect the molecular dynamics, thereby artificially altering the binding event.

MST is a unique physical principle of thermophoresis dependent technique to quantify the interaction between biomolecules 67. Laudably, the sample consumption during the process is much smaller and time saving than previously mentioned methods. Moreover, MST used a label-free method which can avoid anthropogenic influence and increase authenticity 68, 69. In a previous study, the MST technology has been successfully used to detect the affinity of p-PROTAC with gradient dilutions of purified CREPT proteins (K_d_ ≈ 0.34 μM ± 0.11) 18.

Co-IP has recently become one of the most popular assays in PPI research field, and it is often combined with previously mentioned techniques to determine whether two target proteins are bound *in vivo* 70-72. In the Co-IP experiment, the assay begins with the preparation of cell or tissue lysate in an appropriate lysis buffer, and then the POI in the lysate is captured using a specific antibody and precipitated along with its binding proteins with resin 73. It is worth noting that Co-IP cannot be used to detect relatively weak affinity or transient PPI, and the wrong choices of target protein can also lead to the failure of this assay.

### Cell membrane permeability

The cell membrane permeability of fluorescein-labeled p-PROTAC can be assessed using flow cytometry and confocal microscopy. For instance, the fluorescence intensity could be measured to quantify the p-PROTAC within cell membrane using flow cytometry. The specific subcellular localization of FITC or carboxytetramethylrhod-amine (TAMRA) labeled p-PROTAC can be further visualized through co-localization with confocal microscopy 26, 31.

### Bioactivities

In the previous studies, the bioactivities of p-PROTAC were mainly evaluated in the molecular and cellular levels. In the molecular level, western blotting (WB) and quantitative polymerase chain reaction (qPCR) are often used to access the degradation ability of p-PROTAC on POI and related signaling pathway. For example, WB analysis confirmed the degradation of ERα in T47D cells and Tau degradation in multiple cell lines after p-PROTACs treatment, respectively 26, 31. In addition, the mRNA level of pS2, a gene transcriptionally regulated by ERα, was further measured with qPCR analysis for the subsequent downstream effects 31. On the cellular level, the cell viability, cell proliferation, and cell apoptosis are commonly tested to evaluate the efficacy of p-PROTAC 31.

### Ubiquitin-proteasome system-dependent degradation

Once the p-PROTAC ternary complex is successfully formed, ubiquitin proteins will be recruited to POI to initiate the degradation of the pathogenic proteins through the UPS. MG132, the proteasome inhibitor, is often used to verify the specificity of the UPS-dependent degradation caused by p-PROTAC. The previously reported p-PROTACs have exhibited the ability to reduce the expression of POI, such as ERα 31, Tau protein 26, and CREPT 18, which can be antagonized by MG132. Moreover, VHL, an E3 ligase ligand, is also used to verify the specificity of VHL-recruitment in p-PROTAC strategy through preventing recruitment of E3 ligase. The most typical example is that c-Met levels could be rescued after treatment with 50-fold excess free VHL ligand following the PROTAC-7 treatment 78. These functional studies suggested that the degradation through ubiquitin proteasome is the main mechanism of p-PROTAC.

## Application of p-PROTAC

**Figure 3:**
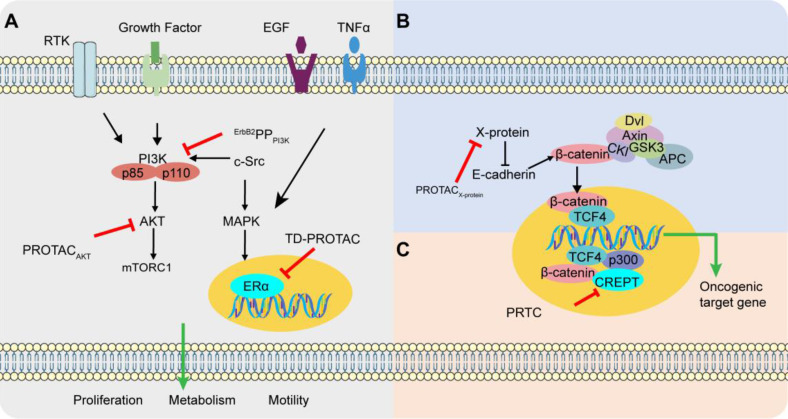
** Different pathways in cancer targeted by p-PROTAC. (A)** ^ErbB2^PP_PI3K_ and PROTAC_AKT_ inhibit breast tumor growth and promote apoptosis through the classic PI3K/AKT signaling pathway. c-Src promotes the transcription of ERα in the nucleus through the MAPK signaling pathway. The degradation of ERα by TD-PROTAC inhibits the transcription of downstream target genes and mediates the killing effect on ERα-positive breast cancer. **(B)** Reduced expression of E-cadherin is closely related to disruption of cell-cell contacts and enhanced cancer cell invasion. When E-cadherin expression is suppressed, β-catenin is released and translocated to the nucleus, which can be antagonized by PROTAC_X-protein_. **(C)** PRTC proficiently inhibits cell proliferation and motility in pancreatic by inhibiting transcription initiation upon Wnt signaling as well as downstream target gene cyclin D1 thus inducing cycle arrest and DNA damage.

The interface of most protein-protein complexes is usually hydrophobic, relatively flat, and lacking deep docking pockets 79. Compared with small molecules, peptide-based modulators exhibit greater potential to regulate PPI because of their large contact surface area and easy modification 40, 80. Therefore, p-PROTAC is a more attracting strategy to eliminate the intracellular pathogenic proteins through UPS dependent degradation cascade. Several p-PROTACs have already been successfully used in some cancers and neurodegenerative disease with the specific POIs, including ERα, PI3K, AKT, CREPT, X-protein, Tau-protein, and FRS2α, which are summarized in **Table 3.** The main signaling pathways targeted by p-PROTACs for tumor treatment are illustrated in **Figure 3.**

### Breast cancer

The ErbB2/ErbB3/PI3K signal transduction pathway plays an important role in mitosis and inhibition of apoptosis 49. Overexpression of ErbB2 in breast and ovarian cancers leads to the formation and phosphorylation of heterodimers, thereby activating the downstream signal transduction 81, 82. In addition, the ErbB3 phosphorylation recruits the lipid kinase PI3K through the SH2 domain of the PI3K regulatory subunit. And the PI3K activation stimulates the survival-promoting kinase Akt (protein kinase B), which inactivates the pro-apoptotic mechanism in tumor cells 49. The successful inhibition of ErbB3/PI3K signaling pathway is emerging as a promising anti-tumor strategy. Recently, a PI3K-targeted phosphor-PROTAC (^ErbB2^PP_PI3K_) has been successfully fabricated to degrade PI3K in breast cancer cell lines MDA-MB-175 and MDA-MB-231 through the inherent affinity of peptide targeting warheads with SH2 domain of PI3K 65, as the first reported p-PROTAC with confirmed *in vivo* activity and no acquired drug-resistant mutations compared with erlotinib and gefitinib 49, 83.

ERα is often overexpressed in breast cancer cells, and promotes estrogen-dependent cell proliferation 84. As the classic methods for inhibiting ERα, modulation of the conformational state of ERα with various unnatural ligands often causes drug resistance in breast cancer patients 85-87. In the previous reported PROTAC, Zhao et al. 30 used the N-terminal aspartic acid to promote formation of helical structures to enhance the stability and cell permeability, which helped the TD strategy-based PROTAC to form a complex with E3 ubiquitin ligase for effective degradation of ERα and inhibition of estrogen-positive breast cancer cell 31. It is worth noting that TD strategy-based p-PTOTAC targeted the different sites of the POI compared with the small molecules PROTAC, and this study proved the successful application of stabilized peptides based PROTAC and its prospects.

### Ovarian cancer

When investigating the effect of p-PROTAC ^ErbB2^PP_PI3K_, authors found that ^ErbB2^PP_PI3K_ could knock out PI3K in two separate ovarian cancer cell lines OVCAR8 and SKOV3 *in vitro* and inhibit tumor growth in OVCAR8 xenograft mouse model 49. In addition, compared with small molecule inhibitor of PI3K (LY294002), the p-PROTAC at dose of 10 mg/kg/day (maximum tolerated dose, MTD) through intraperitoneal injection exhibited lower toxicity 49. In another study, a p-PROTAC (CPP-tria-PR) was prepared to degrade Akt protein *in vitro* which is also closely related with the occurrence of ovarian cancer 33.

### HBV-induced hepatocellular carcinoma (HCC)

The X-protein of hepatitis B virus (HBV) is essential for viral infection and promotes HBV-induced HCC 88-90. A p-PROTAC has been successfully fabricated with an oligomerization peptide, to recruite the E3 ubiquitin ligase for UPS-dependent degradation of X-protein 48.

### Pancreatic cancer

Associated with RNA polymerase II which could induce chromatin loops' formation and activation thus promoting cyclin D1 transcription 91, CREPT is identified as a novel oncogene which is highly expressed in pancreatic cancer 18, 86, 87, 91. Based on this, a cell-permeable p-PROTAC (PRTC), using a peptide binding key in the CCT domain, was fabricated to degrade the CREPT in pancreatic cancer cells 18. These results showed that PRTC is effective in degradation of CREPT proteins, as well as the inhibition effect of tumorigenesis in the tumor bearing xenograft mice without obvious side-effects 18.

### Neurodegenerative diseases

Microtubule-associated protein Tau is clearly clustered in neurodegenerative diseases, which is an ideal target for the treatment of neurodegenerative diseases 92-94. Potential anti-Tau therapies, including kinase inhibition, inhibition of Tau aggregation, or microtubules stabilization, have been discontinued because of the toxicity and/or lack of efficacy 95. In the previous study, a p-PROTAC composed of a peptide from β-tubulin recruiting Tau and a peptide as a strong binder of Keap1 through a short linker has been fabricated. By recruiting Tau to the Keap1-Cul3 ubiquitin ligase complex, the protein Tau was finally degraded through proteasome in several Tau over-expressed cell lines such as SH-SY5Y, Neuro-2a, and PC-12 cells in a concentration-dependent and time-dependent manner 26. Unfortunately, the blood-brain barrier (BBB) permeability of this p-PROTAC was not evaluated.

## Future development

PROTAC technology has achieved notable advances in drug discovery, and several PROTAC molecules are currently in clinical trials 96. There are still many challenges for the future development of PROTAC, including off-target toxicity caused by non-specific degradation of POI in normal cells, major obstacle of pharmacokinetic (PK) evaluation for PROTAC, limited choices of E3 ligases for PROTAC design, and restricted applications for only intracellular proteins but not extracellular proteins, including membrane proteins and secreted proteins. In addition, as one vital form of PROTAC, p-PROTAC further broadens the applicable targets in “undruggable proteins” with potential high affinity (**Table 1**) 3, 25, 31. Nevertheless, p-PROTACs also have their own limitations, including poor structural stability, easy degradation, and poor transmembrane capacity. To fully address those shortcomings, we propose the following possible solutions as shown in **Table 4**, which may be used to tackle the weakness of p-PROTACs thus forwarding their potential clinical translation.

### Utilization of cell-penetrating peptides

The cell-penetrating peptides (CPPs), including HIV-1 TAT peptide (YGRKKRRQRRR) and poly-D-arginine (RRRRRRRR), have already been used to effectively penetrate cell membrane through endocytosis or direct penetration for delivery of many types of cargo, including peptides, proteins, oligonucleotides, and drug molecules. Studies confirmed that these CPPs can also be used for p-PROTACs 49, 97-99. In previous study, two different CPP, poly-D-arginine and TAT peptide, have been used to enhance the Keap1-dependent p-PROTACs for degradation of Tau-protein 26. Through the comparison of EGFP-labelled series of modified p-PROTAC, the poly-D-arginine conjugated p-PROTAC possess the best membrane permeability in SH-SY5Y cells. The similar result can be seen in the poly-D-arginine modified phosphoPROTAC ^TrkA^PP_FRS2α_ and ^ErbB2^PP_PI3K_ with increased inhibitory effects on cells 49. In addition, a new short CPP-Xentry (LCLRPVG), an N-terminal region of the X-protein from hepatitis B virus, has been successfully used in the synthesis of p-PROTAC for X-protein 100.

### Constrained conformation

Besides the cell membrane penetration, there are still some other barriers for improvement of p-PROTAC drug-like characteristics 101. One vital issue is the retention time of the protein epitope mimetic conformation, especially for the short peptides. It is conformational flexibility that causes poor stability and cell permeability of peptides, thereby making intracellular targets inaccessible by originally fragile and hydrophilic peptides 30. Hence, conformational constraint of peptides with unnatural backbone structure provides promising method to solve the previous problems 102-103. Illuminated by nucleation strategies, Li et al. 30 introduced unnatural amino acid 2,3-diaminopropionic acid into PERMs motif backbone to lock its helical conformation. TD strategy-based p-PROTAC (TD-PROTAC) exhibited higher helicity, binding affinities, better stability, and cell permeability than linear PROTACs 31. Another widely applied conformation-constrained strategy for peptide is all hydrocarbon stapled peptide technology developed by Verdine et al 104. This strategy has successfully pushed mimetic peptide-ALRN-6924 as p53-MDM2/MDMX inhibitor into Phase II trial (Clinical Trials.gov Identifier: NCT02264613). Our group has also previously used stapled peptides as PPI inhibitor to regulate Axin-β-catenin interaction with better structural stability, protease resistance and stronger efficacy of Axin (469-482) than linear peptides 102. On this basis, we subsequently reported a new p-PROTAC with stapled technology (xStAx-VHLL) that effectively inhibited Wnt-dependent intestinal cancer in mice and the survival of colorectal cancer patient-derived organoids through degradation of β-catenin 105. These findings strongly corroborate that constrained conformation exhibits the ability to promote the drug-like property of p-PROTAC.

### Targeted delivery

Another key issue is non-specific degradation of POI in normal cells. In this pursuit, the safe biodegradable delivery systems should be proposed for the target delivery of p-PROTAC, such as nanocarriers which have been used to deliver protein or peptide drugs 106-107. While protecting the stability of cargoes, nanomedicine can significantly increase the bioavailability, circulation time, and tissue-specific targeting through surface modification, which can further improve the efficacy 108. For example, PEG conjugated nanoparticle can escape from monocyte phagocytic system uptake, prolonging blood residence time and circulation 109-110. It is also well known that tumor tissues often have enhanced permeability and retention effect (EPR) due to the uncontrolled angiogenesis process and leaky vascular structure 111, and numerous evidences proved the successful utilization of EPR effects for nanomedicine accumulation in tumor sites 112. These findings suggest that the combination of nanotechnology with p-PROTAC strategy may help to solve the difficulties in targeted delivery and specific accumulation of p-PROTAC in tumor sites. For instance, Aronson et al 111 loaded a cytotoxic anti-cancer peptide into the lipid nanoparticles, and this DDS successfully facilitated the selective damage in cancer cells while avoiding off-target effects in normal tissues. Similarly, a serum albumin-coated boehmite nano-DDS for bee venom peptide melittin has also been used to reduce the hemolysis and enhance the cytotoxic effects compared to the free drug 114.

## Conclusion and perspective

The booming p-PROTAC technology offers superior advantages over traditional peptide drugs with auxiliary action mechanism and synergetic therapeutic effect. It also provides great opportunity to drive the further development of existing peptides, especially for PPI inhibitors. At the same time, increasing crystal structure identifications of pathogenic proteins help to accelerate the discovery of specific peptides, making “undruggable” targets potentially accessible by mimetic peptides. So far, a series of studies have proved the successful application of p-PROTAC for POI degradation through ubiquitination.

It is worth noting that, there are still many challenges and limitations for p-PROTACs which could be partially solved through interdisciplinary collaboration including structure biology, chemistry, nanotechnology, and pharmacology. For example, with the emergence of conformational constraint strategy, stabilized peptide-based PROTAC exhibited improved activities, stability, and cell permeability compared to the linear ones. Moreover, we expect that multifunctional drug delivery systems (DDS) can be regarded as novel approaches to improve therapeutic utility of p-PROTAC or achieve combination therapy. Meanwhile, the development of novel preclinical tumor models, including patient-derived organoids (PDO) and patient-derived xenografts (PDX), provides tools to better evaluate the efficacy and side effects of p-PROTACs 105, 115. However, the compositional complexity of p-PROTACs makes it being drug-like more challenge than simple peptides drugs, including the increased technical difficulties in evaluation of drug pharmacokinetics, stability, safety, and especially the unique catalytic cycle manner for p-PROTACs. To better forwarding the future clinical translation of p-PROTACs, experiences learned from the encouraging clinical trials of small molecule PROTACs, including ARV-471 and ARV-110, should be valued.

In general, p-PROTAC technology provides an attractive platform to obtain leading candidates for potential translational research. We expect that the emerging p-PRTOACs will become leading PROTAC modality in the advancement of novel drug discovery.

## Figures and Tables

**Table 1:** Comparison of p-PROTAC and small molecule PROTAC

	p-PROTAC	small molecule PROTAC
Targeting warhead	Peptides	Small molecules
Advantages	Ability to target "undruggable" POIs with specificity 23; Resistance to mutation targets 21; Simple design and synthesis process; Low toxicity and high-safety *in vivo*	High cellular permeability, High stability, and low cost 21
Disadvantages	Poor cell membrane permeability 25; Low stability 25;Few studies on their efficacy	Limitation of degradation of "undruggable" proteins 1; Inability to target "undruggable" with large shallow surfaces 23;Severe side effects
Clinical trials	None	ARV-110, ARV-471

**Table 2:** Characteristics of different binding affinity analysis methods

	FP 64, 74	ITC 75	SPR 66, 76	MST 67, 69	Co-IP 70, 77
Measuring range	pM ~ mM	nM ~μM	pM ~ mM	pM ~ mM	−
Protein fixation	No	No	Yes	No	Yes
Sample consumption	Low	High	Low	Minimal	−
Sample number	1	1	1	1 ~ 16	1
Applicable sample	Proteins; Peptides; Small molecules	Proteins; Peptides; Small molecules	Proteins; Peptides; Small molecules; Cell lysates; Culture medium Viruses, et al.	Proteins; Serum; Cell lysates; Culture medium	Cell lysates; Culture medium
Sensitivity	High	High	High	High	Low
Time-consuming	Hours	Hours	Hours	Minutes	Days
Fluorescent labeling	Yes	Not required	Not required	Yes	No
Advantages	Low sample purity requirements; Real-time monitoring	Low sample purity requirements; Easy to use; Sample could be reused after the test	Real-time monitoring; Wide range of samples; Classic approach	Fast and efficient; Easy to use; Close to the natural testing environment; Reaction system in solution.	Close to the natural testing environment; Commonly used for *in vivo* assay
Disadvantages	Expensive equipment;	Large sample consumption; Kinetics cannot be determined	Non-specific binding; Expensive equipment	Non-specific binding; Kinetics cannot be determined	Low affinity and instant PPI are difficult to detect; the error in predicting the target protein could lead to the failure

**Table 3:** Successful application of p-PROTACs

Name [Ref.]	Sequence	Target	Warhead	E3 ligase	Linker	Cancer
PROTAC_AKT_ 33	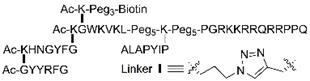	Akt	tri_a	VHL	PEG_5_	Ovarian cancer
TD-PROTAC 31	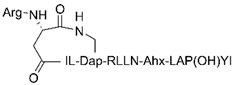	ERα	PERMs	VHL	Ahx	Breast cancer
^ErbB2^PP_PI3K_ 49	GPGGDYAAMGACPASEQGYEEMRA-PEG_3_-ALAPYIP-(D-R)_8_	PI3K	ErbB2 peptide	VHL	PEG_3_	Breast cancer; Ovarian cancer
PRTC 18	KRRRR-VRALKQKYEELKKEK; ESLVDK-Ahx-LAP(OH)YI.	CREPT	CREPT ligand	VHL	Ahx	Pancreatic cancer
PROTAC_X-protein_ 48	(D-R)_8_-LCLRPVGAESRGRPVS; GPFG-GMLAPYIPM.	X-protein	oligomerization peptide	VHL	−	HBV-induced HCC
^TrKA^PP_FRS2α_ 49	IENPQYFSDA-Ahx_2_-ALAPYIP-(D-R)_8_	FRS2α	TrKA phosphorylation site	VHL	Ahx_2_	Neuroma (PC12)
PROTAC _Tau-protein_ 26	YQQYQDATADEQG-GSGS-LDPETGEYL-(D-R)_8_	Tau-protein	Sequence targeting Tau	Keap1	GSGS	Neurodegenerative disease

**Table 4:** Strategies used to improve p-PROTACs

	CPPs 26, 48, 49, 66	Constrained conformation 31	Target delivery
Tools	TAT; poly-D-arginine; Xentry	α-helical conformation of peptides	Nanocarriers
Application	PROTAC_Tau-protein_; ^TrkA^PP_FRS2α_; ^ErbB2^PP_PI3K_; PROTAC_X-protein;_ PRTC	TD-PROTAC	−
Advantages	Increased permeability	Increased permeability and stability	Precise treatment

## Abbreviations

PROTAC: PROteolysis TArgeting Chimera
p-PROTAC: peptide PROTAC
POI: protein of interest
CLL: chronic lymphocytic leukemia
BTK: Bruton's tyrosine kinase
UPS: ubiquitin-proteasome system
ER: estrogen receptor
AR: androgen receptor
BRD4: bromodomain-containing protein 4
ALK: anaplastic lymphoma kinase
CDK9: cyclin dependent kinase 9
CREPT: cycle-related and expression-elevated protein
PPI: protein-protein interaction
PERMs: peptidomimetic estrogen receptor modulators
TD: terminal aspartic acid
VHL: Von Hippel-Lindau
CRBN: cereblon
SCFβ-TRCP: Skp1-Cullin-F boxβ-TRCP
HIF-1α: hypoxia-inducible factor-1α
Ahx: aminohexanoic acid
PEG: polyethylene glycol
TD strategy: N-terminal aspartic acid cross-linking strategy
FP: fluorescence polarization
ITC: isothermal titration calorimetry
SPR: surface plasmon resonance
MST: microscale thermophoresis
Co-IP: co-immunoprecipitation
FITC: fluorescein isothiocyanate
Δ*H*°: molar binding enthalpy
Δ*S*°: binding reaction entropy
TAMRA: carboxytetramethylrhod-amine
WB: western blotting
qPCR: quantitative polymerase chain reaction
^ErbB2^PP_PI3K_: PI3K-targeted phosphor-PROTAC
MTD: maximum tolerated dose
HCC: hepatocellular carcinoma
HBV: hepatitis B virus
BBB: blood-brain barrier
CPPs: cell-penetrating peptides
TD-PROTAC: TD strategy-based p-PROTAC
EPR: enhanced permeability and retention effect
DDS: drug delivery systems
PDO: patient-derived organoids
PDX: patient-derived xenografts

## Authors' Biographic

**Associate Prof. Xin Luan** received his B.Sc. in Pharmaceutics from China Pharmaceutical University (2011). He was awarded his Ph.D. degree in Pharmacology at Shanghai Jiao Tong University (2016), under the supervision of Prof. Hong-Zhuan Chen. He subsequently worked for two years at the University of Michigan under the guidance of Prof. Duxin Sun. His current appointment is Associate Professor at the Shanghai University of Traditional Chinese Medicine (SHUTCM). He is currently working on anti-tumor pharmacology and delivery systems of natural products.

**Prof. Wei-Dong Zhang** obtained his B.Sc. and Master degree in Natural Medicinal Chemistry from Second Military Medical University in 1988 and 1991, respectively. He received his Ph.D. in Natural Medicinal Chemistry from Shanghai Institute of Pharmaceutical Industry (1998), under the supervision of Professor Hui-Ting Li. He is currently a professor of the SHUTCM and Second Military Medical University. His research mainly focuses on Chinese medicine formula, isolation, structural identification and modification, total synthesis, and structure-activity relationship of bioactive natural products.

**Jinmei Jin** obtained her Master's degree (2019) in institute of Chinese Materia Medica at SHUTCM. She is currently a Ph.D. student under the supervision of Prof. Hong-Zhuan Chen and Xin Luan. Her current research focuses on the synthesis of peptide PROTAC and related oncotherapy.

**Ye Wu** obtained his B.Sc. in Pharmaceutics (2013) and Master degree in Pathology and Pathophysiology (2017) at Chengdu Medical College. He is currently pursuing his Ph.D. studies at SHUTCM, under the supervision of Prof. Wei-Dong Zhang and Xin Luan. His research mainly focuses on the conformational constraint and targeted delivery of natural cytotoxic peptides.

**Jinjiao Chen** obtained her bachelor's degree from the School of Life Science and Medicine, Dalian University of Technology in 2015. She is currently studying as a combined training student of Fudan University and SHUTCM, under the guidance of Prof. Xue-Mei Zhang and Xin Luan. Her research focuses on the development of anti-tumor proteolysis targeting chimera compounds.
